# Potential causal association of diabetes mellitus and blood glucose related indexes with the onset of epilepsy: a two-sample Mendelian randomization study

**DOI:** 10.3389/fneur.2024.1399504

**Published:** 2024-06-19

**Authors:** Mengting Zhu, Shuying Ling

**Affiliations:** Department of Neurology, The Fifth People's Hospital of Wujiang District, Suzhou, Jiangsu, China

**Keywords:** diabetes mellitus, blood pressure, epilepsy, Mendelian randomization study, causal association

## Abstract

**Aim:**

Diabetes mellitus (DM) may promote the occurrence of epilepsy through mechanisms, such as inflammation, immune imbalance, and cerebrovascular injury, caused by metabolic abnormalities. However, evidence for the effects of DM and blood glucose (BG) on the risk of epilepsy is limited. Herein, this study used the Mendelian randomization (MR) method to investigate the potential causal associations of DM and BG-related indexes with epilepsy.

**Methods:**

In this two-sample MR study, summary statistics data of the genome-wide association studies (GWASs) on exposures, including type 1 diabetes mellitus (T1DM), T2DM, fasting glucose, and glycated hemoglobin (HbAlc), were extracted from the MRC-Integrative Epidemiology Unit (MRC-IEU). The GWAS data on study outcomes, including epilepsy, focal epilepsy, and generalized epilepsy, were obtained from the FinnGen consortium. MR-Egger regression was used to examine horizontal pleiotropism of instrumental variables (IVs), and Cochran's Q statistics was used to quantify the heterogeneity. MR analysis methods including inverse variance weighted (IVW) tests, weighted median, and MR-Egger were utilized to investigate the causal associations between DM and BG-related indexes with epilepsy. The evaluation indexes were odds ratios (ORs) and 95% confidence intervals (CIs). Reverse causal association analyses were also performed. In addition, IVW-radial and leave-one-out tests were utilized for sensitivity analyses.

**Results:**

IVW estimates suggested that T1DM has potential causal associations with epilepsy (OR = 1.057, 95% CI: 1.031–1.084) and generalized epilepsy (OR = 1.066, 95% CI: 1.018–1.116). No significant reverse causal associations of T1DM with epilepsy or generalized epilepsy were found (all *P* > 0.05). In addition, sensitivity analysis results identified no outlier, indicating that the associations of T1DM with epilepsy and generalized epilepsy were relatively robust.

**Conclusion:**

Patients with T1DM had a potential risk of developing epilepsy, and prompt treatment of DM and dynamic monitoring may be beneficial to prevent epilepsy in this high-risk population. However, the causal associations of DM and BG with epilepsy may warrant further verification.

## Introduction

Epilepsy is one of the most common serious diseases of the central nervous system (CNS) affecting more than 70 million people worldwide (1). Although antiepileptic drugs can suppress up to two-thirds of seizures, they do not change the long-term prognosis, and even patients who do not have seizures often experience adverse drug reactions (2, 3). Therefore, from the perspective of primary prevention, identifying modifiable risk factors is essential to prevent the onset of epilepsy and reduce the disease burdens.

Diabetes mellitus (DM), including type 1 diabetes mellitus (T1DM) and T2DM, is a chronic metabolic disease characterized by high blood glucose (BG), caused by complete/partial insufficiency of insulin secretion and insulin action, which is a major health problem contributing to the global burden of disease (4, 5). Recent evidence suggested DM may promote the occurrence of epilepsy and other neurological diseases through mechanisms, such as inflammation, immune imbalance, and cerebrovascular injury, caused by metabolic abnormalities (6–8). Several cohort studies have shown that patients with T1DM and T2DM have a significantly increased risk of subsequent epilepsy compared with non-T1DM and non-T2DM and that severe hypoglycemia may also increase the risk of epilepsy (9–12). However, evidence for the effects of DM and BG on the risk of epilepsy is still limited at present. In addition, traditional epidemiological studies are susceptible to confounding factors and causal inversion that the true associations of DM and BG with the onset of epilepsy are unclear.

Mendelian randomization (MR) is a burgeoning approach that exploits single nucleotide polymorphisms (SNPs) as unconfounded instrumental variants (IVs) to investigate the potential causal associations between exposures and diseases (13). Meanwhile, genetic code is not affected by environmental factors or preclinical diseases, thus MR analysis is less susceptible to bias resulting from reverse causation. More recently, MR analysis has also been utilized to investigate the potential causes of epilepsy (14, 15). Nevertheless, no study has discussed the causal associations of DM or BG with the onset of epilepsy based on MR methods.

In this study, we conducted a two-sample MR study to explore the potential causal associations between DM and BG-related indexes with epilepsy, aiming to provide an evidence-based foundation for the prevention of epilepsy and screening for high-risk populations.

## Methods

### Data sources

Genome-wide association study (GWAS) data on study exposures, including T1DM, T2DM, fasting glucose, and glycated hemoglobin (HbAlc), were extracted from the MRC Integrative Epidemiology Unit (MRC-IEU) (https://gwas.mrcieu.ac.uk/), and those on study outcomes (including epilepsy, focal epilepsy, and generalized epilepsy) were obtained from the FinnGen consortium (https://www.finngen.fi/fi.). The summarization and presentation of the aggregated information on the data source are shown in Table 1. The involved GWASs have been approved by the respective institutions. Data in each GWAS are de-identified, and informed consent from participants has been obtained. Since the databases were publicly available, ethical approval has been waived by our institutional review board (IRB).

**Table 1:** Aggregated data of study exposures and outcomes.

**GWAS ID**	**Trait**	**Year**	**Sample size**	**Population**	**Consortium\PMID**
**Exposures**					
ebi-a-GCST010681	T1DM	2020	24,840	European	32005708
ebi-a-GCST007515	T2DM	2018	298,957	European	29632382
ebi-a-GCST90002232	Fasting glucose	2021	200,622	European	34059833
ebi-a-GCST90002244	HbAlc	2021	146,806	European	34059833
**Outcomes**					
finn-b-G6_EPLEPSY	Epilepsy	2021	182,367	European	FINNGEN
finn-b-FE	Focal epilepsy	2021	213,461	European	FINNGEN
finn-b-GE	Generalized epilepsy	2021	214,313	European	FINNGEN

T1DM, type 1 diabetes mellitus; T2DM, type 2 diabetes mellitus; HbAlc, glycated hemoglobin.

### Selection of SNPs

Potential IVs were SNPs that were significantly associated with exposure and were selected using the threshold of *P* < 5.0 × 10^−8^ (16). According to the MR principle to ensure the same allele corresponds to the effects between SNPs and the exposure and on the outcomes, SNPs with linkage disequilibrium (LD) and being palindromic with intermediate allele frequencies were removed (the threshold was set to *r*^2^ = 0.001, and the clumping distance was 10,000 kb).

### The assumptions of MR analysis

MR analysis should conform to three assumptions to minimize the impact of bias. First, IVs are associated with exposures and outcomes independent of confounders. The association strength of exposure with IVs was estimated by the formula: *F* = *r*^2^^*^ (*N*−2)/(1–*r*^2^), *r*^2^ = 2 ^*^ EAF ^*^ (1–EAF) ^*^ *b*^2/^SD^2^, in which *N* represented sample size, EAF was the effect allele frequency, *b* represented the regression coefficient for exposure and IVs, and SD was the standard difference. There is a weak association between IVs and exposure if *F* < 10. Second, the IVs must be significantly associated with the exposure. To monitor the potential horizontal pleiotropy effect, that is, the confounding effect caused by other diseases, the MR-Egger regression test was utilized to determine whether it violated the MR second assumption (17, 18). The existence of pleiotropy was represented by the significant intercept item of MR-Egger analysis. Third, IVs affect outcome only through exposure, that is, there is no horizontal pleiotropy effect of IVs on the outcome.

### Statistical analysis

The potential causal associations of DM and BG with epilepsy were estimated through the inverse variance weighted (IVW) method, which is the primary approach to calculate the unbiased estimates of causal effect when horizontal pleiotropy was absent. The weighted-median method can provide a robust and consistent estimate even if nearly 50% of genetic variants were invalid instruments. MR-Egger regression's intercept can examine the presence of potential pleiotropy in IVs (*P* > 0.05 represents no horizontal pleiotropy). In addition, robust adjusted profile score (MR-RAPS) can provide robust estimates in the presence of systematic and idiosyncratic pleiotropy. The evaluation indexes were odds ratios (ORs) and 95% confidence intervals (CIs). The potential causal relationships were statistically significant when *P* < 0.05. The Cochrane's *Q*-test was utilized for heterogeneity test, and IVs with *P* < 0.05 were recognized as heterogeneous. In addition, sensitivity analysis was performed by IVW-radial and leave-one-out methods. Statistical analyses were performed using R version 4.2.0 (Institute for Statistics and Mathematics, Vienna, Austria) with the R package “TwoSampleMR”.

## Results

### Instrumental variables selection

**Figure 1:**
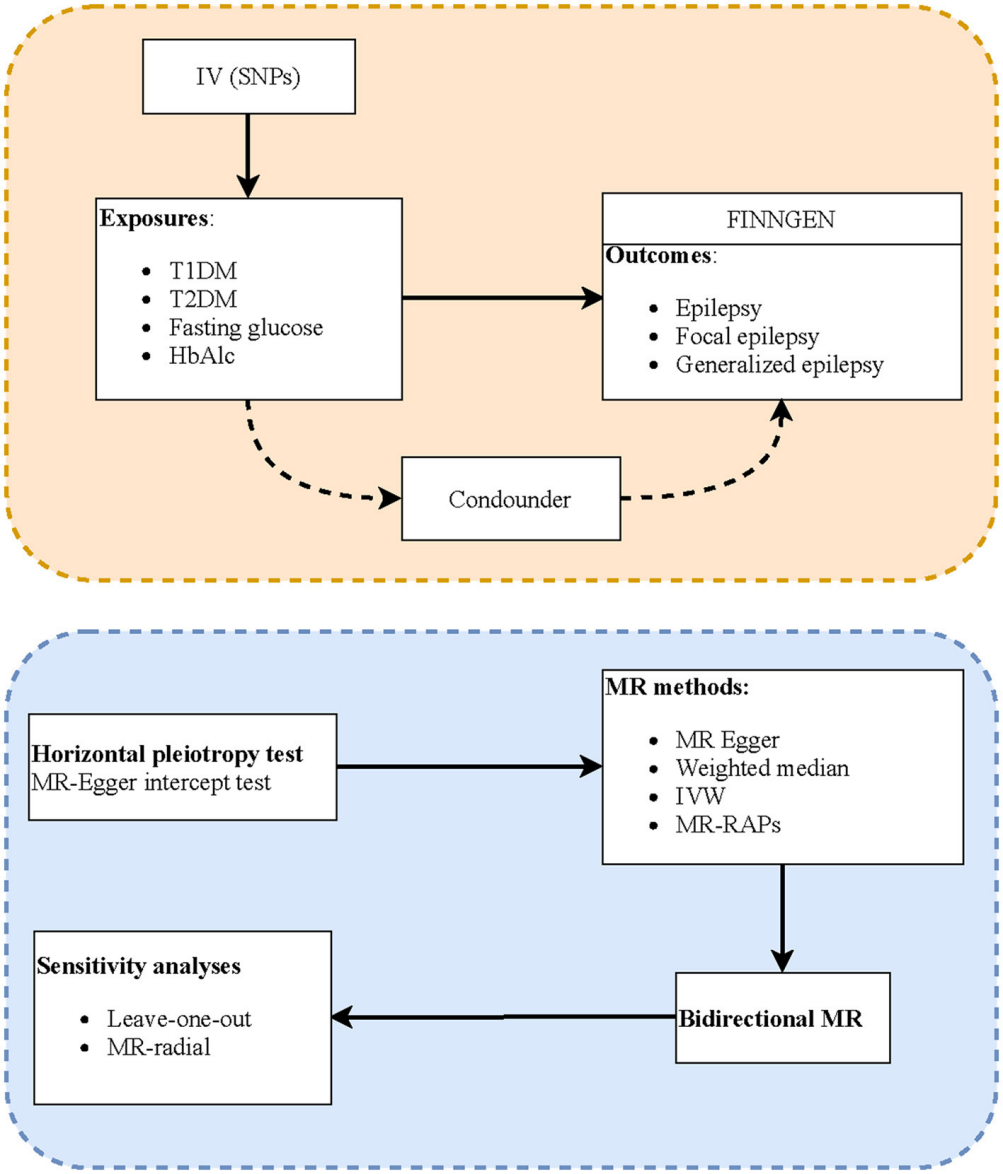
Flowchart of the study process.

Figure 1 shows the study process, and Table 2 shows the selection of IVs. We identified 6,098 SNPs as potential IVs for T1DM, 8,581 for T2DM, 20,604 for fasting glucose, and 221 for HbAlc. After omitting LD SNPs, the numbers of SNPs were 44, 67, 66, and 74, respectively. Then all palindromic or incompatible SNPs were deleted, and the numbers of eligible IVs were 28, 61, 59, and 65, respectively.

**Table 2:** Selection of IVs associated with exposures.

**Exposures**	**Selected SNPs (*P* < 5 × 10^−8^)**	**Omitted LD SNPs**	**Drop all palindromic SNPs**
T1DM	6,098	44	28
T2DM	8,581	67	61
Fasting glucose	20,604	66	59
HbAlc	221	74	65

IV, instrumental variant; SNP, single nucleotide polymorphisms; LD, linkage disequilibrium; T1DM, type 1 diabetes mellitus; T2DM, type 2 diabetes mellitus; HbAlc, glycated hemoglobin.

Next, the horizontal pleiotropy, heterogeneity, and strength of association were evaluated (Table 3). The selected IVs were associated with exposures, with *F*-values > 10. No horizontal pleiotropy was found according to the MR-Egger regression results (all *P* > 0.05). However, there was heterogeneity between IVs that were associated with fasting glucose and focal epilepsy (MR-Egger *Q* = 79.0295, *P* = 0.0284; IVW *Q* = 81.0381, *P* = 0.0246).

**Table 3:** Test for strength, horizontal pleiotropy, and heterogeneity.

**Variables**	**Strength**	**Horizontal pleiotropy test**		**Heterogeneity test**			
	***F***, *R*^2^	**MR-Egger intercept**	***P***	**MR-Egger** ***Q***	***P***	**IVW** ***Q***	***P***
**Epilepsy**							
T1DM	40.459, 0.161	0.0015	0.8299	24.3752	0.5545	24.4223	0.6068
T2DM	33.841, 0.011	0.0037	0.595	67.0296	0.221	67.3541	0.2401
Fasting glucose	54.97, 0.027	0.0082	0.174	58.779	0.4101	60.7329	0.3777
HbAlc	34.378, 0.023	−0.0023	0.7058	81.6014	0.0576	81.7876	0.0663
**Focal epilepsy**							
T1DM	40.459, 0.161	0.0015	0.9351	26.9433	0.4123	26.9503	0.4665
T2DM	33.841, 0.011	0.0176	0.2857	57.0802	0.5466	58.241	0.5403
Fasting glucose	54.97, 0.027	0.0209	0.2337	79.0295	**0.0284**	81.0381	**0.0246**
HbAlc	34.378, 0.023	−0.0041	0.7695	67.9049	0.3138	67.9983	0.3428
**Generalized epilepsy**							
T1DM	40.459, 0.161	0.0222	0.1217	30.3232	0.2544	33.3084	0.1871
T2DM	33.841, 0.011	0.0103	0.3853	52.2596	0.7203	53.0246	0.7263
Fasting glucose	54.97, 0.027	0.0103	0.337	36.4496	0.9845	37.387	0.9838
HbAlc	34.378, 0.023	−0.0126	0.2418	74.1395	0.1592	75.7824	0.1488

*F* = *r*^2^^*^ (*N*−2)/(1–*r*^2^), *r*^2^ = 2 ^*^ EAF ^*^ (1–EAF) ^*^ *b*^2^/SD^2^, MR, Mendelian randomization; IVW, inverse variance weighted; T1DM, type 1 diabetes mellitus; T2DM, type 2 diabetes mellitus; HbAlc, glycated hemoglobin. Bold values represented exposure and outcome related SNPs have heterogeneity.

### Potential causal associations of DM and BG-related indexes with epilepsy

Table 4 shows the associations of DM and BG-related indexes with epilepsy. IVW estimates showed there were potential causal associations of T1DM with both epilepsy (OR = 1.057, 95% CI: 1.031–1.084) and generalized epilepsy (OR = 1.066, 95% CI: 1.018–1.116), indicating that patients with T1DM had an increased odds of 0.057 of epilepsy and 0.066 of generalized epilepsy. In addition to the IVW, MR-Egger (OR = 1.054, 95% CI: 1.016–1.093), weighted median (OR = 1.050, 95% CI: 1.017–1.084), and MR-RAPS (OR = 1.027, 95% CI: 1.007–1.047) methods were also suggested a potential causal association of T1DM with epilepsy. Moreover, as shown in Table 5, no significant reverse causal associations of T1DM with epilepsy were found (all *P* > 0.05), which indicated that the association of T1MD with epilepsy or with generalized epilepsy was unidirectional.

**Table 4:** Potential causal associations of DM and BG-related indexes with epilepsy.

**Variables**	**Methods**	**OR (95% CI)**	***P***
**Epilepsy**			
T1DM	MR-Egger	1.054 (1.016–1.093)	**0.0092**
	Weighted median	1.050 (1.017–1.084)	**0.0025**
	IVW	1.057 (1.031–1.084)	**1.23** **×10**^**−5**^
	MR-RAPS	1.027 (1.007–1.047)	**0.0076**
T2DM	MR-Egger	0.971 (0.788–1.196)	0.7800
	Weighted median	1.117 (0.981–1.273)	0.0957
	IVW	1.022 (0.941–1.110)	0.6052
	MR-RAPS	1.014 (0.937–1.097)	0.7287
Fasting glucose	MR-Egger	0.724 (0.417–1.257)	0.2565
	Weighted median	0.941 (0.616–1.438)	0.7789
	IVW	1.015 (0.777–1.326)	0.9147
	MR-RAPS	1.139 (0.905–1.432)	0.2671
HbAlc	MR-Egger	0.981 (0.466–2.066)	0.9606
	Weighted median	0.840 (0.486–1.454)	0.5340
	IVW	0.868 (0.616–1.223)	0.4185
	MR-RAPS	0.952 (0.682–1.330)	0.7741
**Focal epilepsy**			
T1DM	MR-Egger	1.038 (0.945–1.140)	0.4480
	Weighted median	1.070 (0.987–1.159)	0.0986
	IVW	1.041 (0.977–1.108)	0.2142
	MR-RAPS	1.044 (0.993–1.096)	0.0892
T2DM	MR-Egger	0.808 (0.492–1.325)	0.4006
	Weighted median	0.991 (0.723–1.358)	0.9536
	IVW	1.034 (0.839–1.273)	0.7562
	MR-RAPS	1.059 (0.867–1.293)	0.5736
Fasting glucose	MR-Egger	0.516 (0.103–2.596)	0.4258
	Weighted median	0.812 (0.279–2.360)	0.7015
	IVW	1.224 (0.551–2.720)	0.6198
	MR-RAPS	1.126 (0.632–2.008)	0.6872
HbAlc	MR-Egger	1.033 (0.186–5.731)	0.9708
	Weighted median	0.679 (0.175–2.637)	0.5755
	IVW	0.829 (0.349–1.971)	0.6720
	MR-RAPS	0.932 (0.401–2.166)	0.8692
**Generalized epilepsy**			
T1DM	MR-Egger	1.020 (0.949–1.097)	0.5881
	Weighted median	1.057 (0.998–1.119)	0.0568
	IVW	1.066 (1.018–1.116)	**0.0063**
	MR-RAPS	1.032 (0.996–1.069)	0.0854
T2DM	MR-Egger	0.929 (0.649–1.330)	0.6897
	Weighted median	1.026 (0.795–1.323)	0.8460
	IVW	1.074 (0.924–1.249)	0.3513
	MR-RAPS	1.048 (0.907–1.211)	0.5250
Fasting glucose	MR-Egger	0.806 (0.298–2.176)	0.6718
	Weighted median	1.282 (0.563–2.919)	0.5541
	IVW	1.235 (0.757–2.014)	0.3973
	MR-RAPS	1.333 (0.876–2.028)	0.1796
HbAlc	MR-Egger	1.527 (0.416–5.601)	0.5259
	Weighted median	1.260 (0.464–3.421)	0.6497
	IVW	0.783 (0.418–1.466)	0.4447
	MR-RAPS	0.951 (0.516–1.753)	0.8723

DM, diabetes mellitus; BG, blood glucose; OR, odds ratio; CI, confidence interval; T1DM, type 1 diabetes mellitus; MR, Mendelian randomization; IVW, inverse variance weighted; T2DM, type 2 diabetes mellitus; HbAlc, glycated hemoglobin. Bold values represented the causal association between exposure and outcome is statistically significant.

**Table 5:** Reverse causal associations between T1DM and epilepsy.

**Variables**	**SNPs**	**Methods**	**OR (95% CI)**	***P***
Epilepsy	8	MR-Egger	0.992 (0.899–1.094)	0.8704
	8	Weighted median	0.992 (0.895–1.100)	0.8779
	8	IVW	0.994 (0.917–1.077)	0.8802
	8	MR-RAPS	0.994 (0.912–1.083)	0.8865
Focal epilepsy	13	MR-Egger	1.027 (0.949–1.110)	0.5572
	13	Weighted median	1.016 (0.961–1.074)	0.5712
	13	IVW	1.015 (0.960–1.072)	0.6066
	13	MR-RAPS	1.015 (0.970–1.061)	0.5210
Generalized epilepsy	5	MR-Egger	1.005 (0.937–1.079)	0.8845
	5	Weighted median	0.989 (0.919–1.065)	0.7778
	5	IVW	1.005 (0.954–1.059)	0.8586
	5	MR-RAPS	1.006 (0.953–1.061)	0.8363

T1DM, type 1 diabetes mellitus; SNP, single nucleotide polymorphisms; OR, odds ratio; CI, confidence interval; MR, Mendelian randomization; IVW, inverse variance weighted.

### Sensitivity analysis

**Figure 2:**
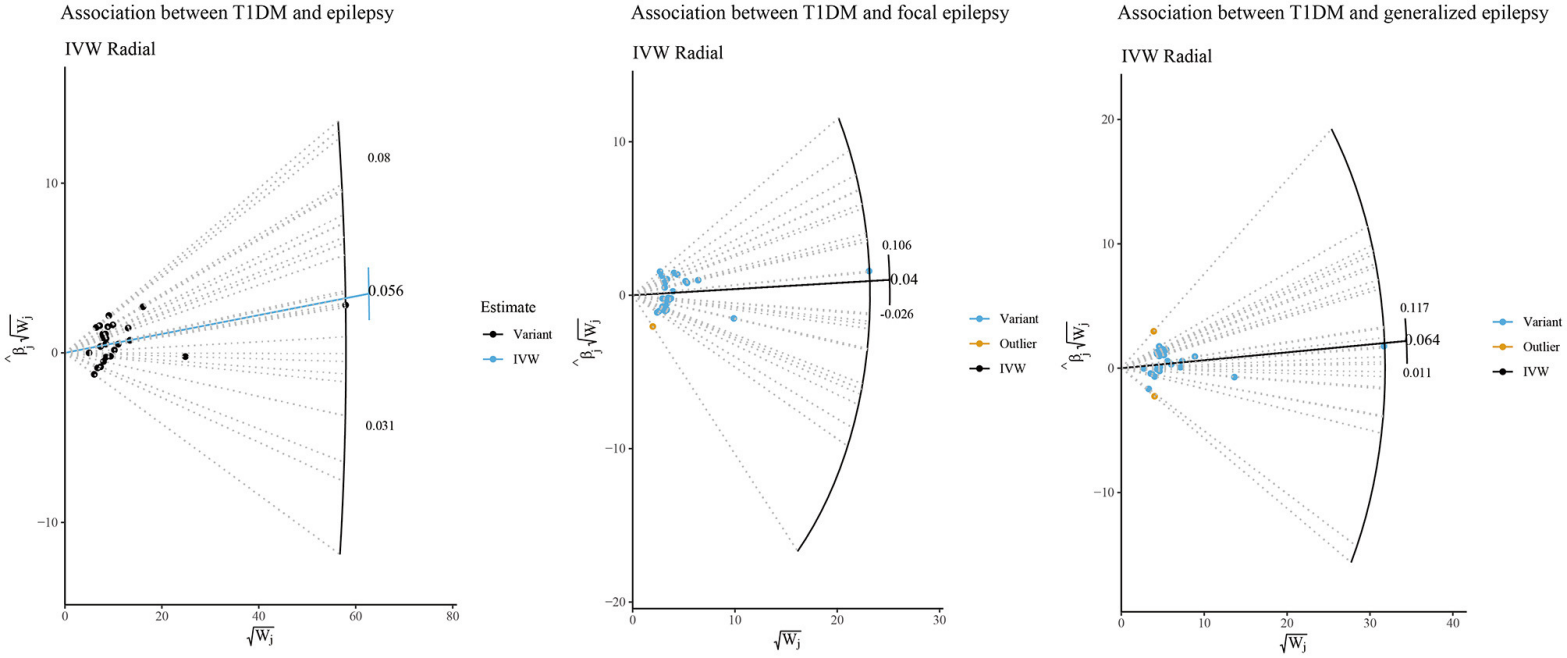
IVW-radial sensitivity analysis of potential causal associations of T1DM with epilepsy, focal epilepsy, and generalized epilepsy.

**Figure 3:**
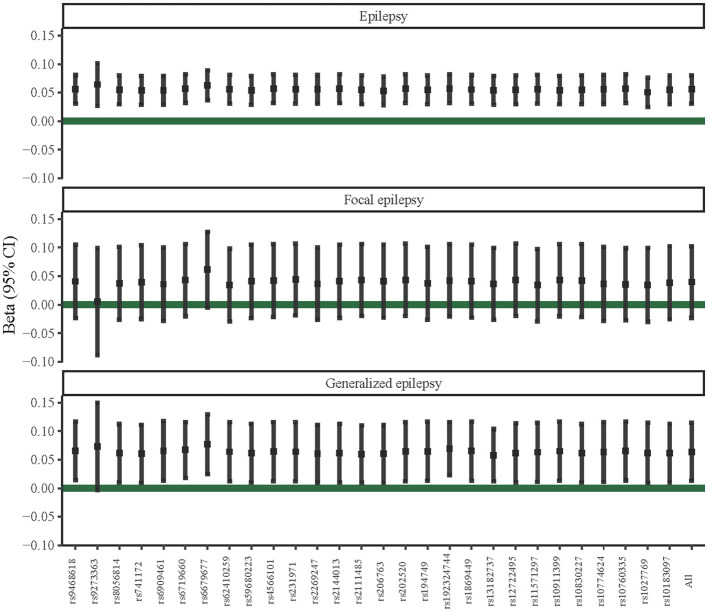
Leave-one-out sensitivity analysis of potential causal associations of T1DM with epilepsy, focal epilepsy, and generalized epilepsy.

We further performed sensitivity analyses of the potential causal associations of T1DM with epilepsy, focal epilepsy, and generalized epilepsy. According to Figure 2, the IVW-radial test identified outliers (yellow points in the plots) in the association between T1DM and focal epilepsy and between T1DM and generalized epilepsy, respectively, rather than that between T1DM and epilepsy. This suggested the potential causal association of T1DM with epilepsy was not influenced by outliers, that is, it was relatively robust. Similarly, the forest plots of the leave-one-out test showed there was no outlier in the association between T1DM and epilepsy (Figure 3). In addition, among relationships between T1DM-related IVs and focal epilepsy or generalized epilepsy, the IV with ID of rs9273363 had a 95% CI including “0”, indicating these causal associations were not steady.

## Discussion

This two-sample MR analysis explored the potential causal associations between DM and BG-related indexes with epilepsy. The study results showed that patients with T1DM seemed to have a potential risk of developing epilepsy and generalized epilepsy. In addition, no reverse causal association was found between T1DM and epilepsy or generalized epilepsy.

To the best of our knowledge, this is the first study to discuss potential causal associations between DM and BG with epilepsy based on the MR approach. Some observational studies have explored the relationship between DM and subsequent epilepsy risk, which may provide some pathological evidence for further causal association investigation, but the causal inference is limited. A meta-analysis conducted involved the aggregation of data from ten datasets, which were obtained from six observational studies and found that individuals with T1DM exhibited a significantly heightened susceptibility to epilepsy when compared with those with normoglycemia (19). Falip et al. (20) recruited adult onset T1DM patients who were seen consecutively in the Diabetes Unit of the Hospital Universitari de Bellvitge, calculated the prevalence of epilepsy and/or seizures, and showed that patients with high titers of glutamic acid decarboxylase antibody were more likely to develop temporal lobe epilepsy. A population-based cohort study of Danish citizens suggested T2DM was associated with several psychiatric and neurological disorders, including epilepsy, and most associations were consistently found for both temporal order of disorders (9). In addition, another cohort study included patients with T2DM and matched controls in Taiwan in 2002–2003, following the incidence of epilepsy or by the end of 2011, which supported that severe hypoglycemia may increase the risk of epilepsy and that T2DM increased the risk of epilepsy independent of severe hypoglycemia (10). In the current study, although no significant association was found between T2DM or BG with epilepsy, similar to Falip's results, T1DM had a potential unidirectional causal association with epilepsy and generalized epilepsy. MR design can make causal inferences compared with previous observational studies so that our findings relatively provided some reference for further clarification on the true association between DM and epilepsy. Nevertheless, the study population of this MR research was only European, and due to the limitation of the GWAS database, we could not obtain data on the exact location of epilepsy as well. Therefore, further studies are still needed to reveal correlations between DM and BG with epilepsy onset risk.

Several studies have explored the etiology of epilepsy, from which we can speculate about the possible mechanisms of the association between T1DM and epilepsy. T1DM is a T-cell-mediated autoimmune disease whose etiology is not fully understood. During T1DM pathogenesis, mitochondrial dysfunction due to impaired mitophagy with the release of reactive oxygen species contributes to initiating an inflammatory response by elevating pro-inflammatory cytokines (21). Meanwhile, clinical and animal studies have shown a complex role of inflammation in the development and progression of epilepsy (22). An elevated inflammatory response increases the secretion of pro-inflammatory cytokines and markers of inflammation and impairs neural circuits (23, 24). Recently, an MR study conducted by Sun et al. (25) showed that three inflammatory cytokines were associated with epilepsy, five were associated with generalized epilepsy, and four were linked to focal epilepsy. Herein, inflammation plays a key role in the underlying link between T1DM and epilepsy. Besides, hypothalamus-pituitary-adrenal (HPA) axis dysfunction may also be involved in the pathway that DM had a causal association with epilepsy. Patients with T2DM showed an impaired cortisol or growth hormone response, which is mainly regulated through the CNS (26, 27). Circadian rhythms of plasma cortisol concentrations instructed by the HPA axis may alter the balance between neuronal excitability and inhibition and modify neuronal excitability and epilepsy through gamma-aminobutyric acid levels (28, 29). However, more evidence should be proposed to clarify the exact mechanisms of the causal association between T1DM and epilepsy in the future.

MR is a relatively superior study design to observational studies on inferring the causal effect of potential risk factors on diseases of interest as mentioned before. Through investigating the roles of DM and BG in epilepsy risk, the present MR study may facilitate the recommendation of public health policies and clinical interventions that effectively reduce the incidence and social burden of epilepsy among the European population. We performed reverse causal association analysis and sensitivity analyses of significant results and found there was no reverse causal association of T1DM with epilepsy, focal epilepsy, or generalized epilepsy. In addition, although the causal association between BG and epilepsy was not significant, in fact, seizures usually improve with the control of glycaemic status in patients with T1DM and T2DM (30). Therefore, in clinical practice, the BG fluctuations need to be reduced timely among both patients with DM and those who had abnormal BG indexes, and at the same time, symptomatic treatment must be administered to avoid severe hypoglycemia or hyperglycemia, thereby reducing the potential risk of epilepsy.

This two-sample MR research explored the causal associations between DM and BG with epilepsy, which could overcome the influence resulting from common confounding factors and reverse causal association. Based on publicly available large-sample GWASs, IVs associated with study exposures and outcomes were highly explanatory and representative. In addition, sensitivity analyses were carried out by IVW-radial and leave-one-out methods, and the results indicated the potential causal association between T1DM and epilepsy was relatively robust. However, there are still some limitations that limited the interpretation of study results. Involved GWASs only included the European population, which limited these potential causal associations extrapolated to other populations. Aggregated data of GWASs limited further exploration of the potential causal associations of DM and BG with epilepsy in individuals with different characteristics. In addition, lacking individual data prevented the assessment of potential non-linear associations between them.

## Conclusion

T1DM had a potential causal association with epilepsy. Patients with DM should be treated symptomatically and monitored timely to reduce the potential risk of epilepsy in clinical practice. However, the true relationship between DM and epilepsy needs to be further clarified.

## Data availability statement

The datasets presented in this study can be found in online repositories. The names of the repository/repositories and accession number(s) can be found in the article.

## Ethics statement

Ethical review and approval was not required for the study on human participants in accordance with the local legislation and institutional requirements. Written informed consent from the patients/participants or patients/participants' legal guardian/next of kin was not required to participate in this study in accordance with the national legislation and the institutional requirements.

## Author contributions

MZ: Conceptualization, Methodology, Project administration, Supervision, Writing – original draft, Writing – review & editing. SL: Data curation, Formal analysis, Investigation, Methodology, Writing – review & editing.
